# Metabolic Variations, Antioxidant Potential, and Antiviral Activity of Different Extracts of *Eugenia singampattiana* (an Endangered Medicinal Plant Used by Kani Tribals, Tamil Nadu, India) Leaf

**DOI:** 10.1155/2014/726145

**Published:** 2014-07-15

**Authors:** K. M. Maria John, Muniappan Ayyanar, Subbiah Jeeva, Murugesan Suresh, Gansukh Enkhtaivan, Doo Hwan Kim

**Affiliations:** ^1^Department of Bioresources and Food Science, Konkuk University, Seoul 143-701, Republic of Korea; ^2^Department of Botany & Microbiology, AVVM Sri Pushpam College (Autonomous), Poondi, Thanjavur District, Tamil Nadu 613503, India; ^3^College of Veterinary Medicine and Veterinary Science Research Institute, Konkuk University, 120 Neungdong-ro, Gwangjin-gu, Seoul 143-729, Republic of Korea; ^4^Department of Botany, VHN Senthikumara Nadar College, Virudhunagar, Tamil Nadu 626001, India

## Abstract

*Eugenia singampattiana* is an endangered medicinal plant used by the Kani tribals of South India. The plant had been studied for its antioxidant, antitumor, antihyperlipidemic, and antidiabetic activity. But its primary and secondary metabolites profile and its antiviral properties were unknown, and so this study sought to identify this aspect in *Eugenia singampattiana* plant through different extraction methods along with their activities against porcine reproductive and respiratory syndrome virus (PRRSV). The GC-MS analysis revealed that 11 primary metabolites showed significant variations among the extracts. Except for fructose all other metabolites were high with water extract. Among 12 secondary metabolites showing variations, the levels of 4-hydroxy benzoic acid, caffeic acid, rutin, ferulic acid, coumaric acid, epigallocatechin gallate, quercetin, myricetin, and kaempferol were high with methanol extract. Since the flavonoid content of methanol extracts was high, the antioxidant potential, such as ABTS, and phosphomolybdenum activity increased. The plants antiviral activity against PRRSV was for the first time confirmed and the results revealed that methanol 25 *µ*g and 75 to 100 *µ*g in case of water extracts revealed antiviral activity.

## 1. Introduction

*Eugenia singampattiana *Bedd. is a small tree belonging to the family Myrtaceae. The plant is endemic to the dry evergreen forests of Agasthiya Hills in southern Western Ghats, India. It is restricted to Singampatti and Papanasam forests of Agasthiya hills, Tamil Nadu, India. The plant was first reported by Beddome during 1864–74. After more than 112 years it was collected and reported [1]. Sarcar et al. [1] assessed the total number of individuals in two isolated populations and stated that the species has narrow zones of endemism and the species is positioned as Critically Endangered in the recent list of IUCN Red List of Threatened Species [2].

*E. singampattiana* is locally (Tamil) known as Kaattukorandi and reported to have several medicinal properties. Kani tribal people in Agasthiya Hills of Tamil Nadu use the powdered leaves of* E. singampattiana* for the treatment of constipation and to strengthen the body [3]. Sutha et al. [4] reported that the powdered leaves of* E. singampattiana* are consumed to treat rheumatism by the Kani tribals. The paste made from the leaves of* E. singampattiana* is used to treat asthma, giddiness, body pain, throat pain, leg sores, rheumatism, and gastric complaints [5]. Leaf extracts of* E. singampattiana* is also reported for various biological activities such as antitumour [6], antioxidant, antihyperlipidemic, antidiabetic [7], and hepatoprotective [8] activity.

Medicinal plants are one of the rich sources for the development of new pharmaceutical products; hence, these plants are rich in secondary metabolites. Studies on metabolic diversity and their effect on various diseases by* in vitro* methods will be helpful for the discovery of new therapeutics.* E. singampattiana* was traditionally used by the tribal community of South India, but their activity against viral diseases is not scientifically studied. Moreover the terpenoids and ketones were listed by Kala et al. [9] but the phenolic compound diversity particularly the flavonoids was unknown.

## 2. Materials and Methods

### 2.1. Collection of* Eugenia singampattiana*

Leaf samples of* E. singampattiana* are collected from the forests of Karayar region in Agasthiya Hills of southern Western Ghats, Tamil Nadu, India. The voucher specimens of* E. singampattiana* (SPCH-MA 94) are deposited in the herbarium of A.V.V.M. Sri Pushpam College Herbarium, Poondi, Tamil Nadu, India, for future reference. The leaves were collected, shade-dried, and used for the extraction. Since this plant belongs to endemic, less number of leaf samples were collected and used for the analysis.

### 2.2. Different Solvent Extraction for Biochemical Screening

Ethanol (ESE), methanol (ESM), and hot water (ESW) were used as solvents for the extraction of metabolites from* E. singampattiana* leaves. 2 g of powdered leaf samples was extracted with 20 mL of ESE, ESM, and ESW three times and was centrifuged at 8000 rpm. The supernatants were collected and evaporated to dryness using a rotary evaporator. The residues from the ESE, ESM, and ESW were redissolved with distilled water (10 mL) separately and were filtered. In case of ethyl acetate extraction, 2 g of leaf samples was extracted with 20 mL of hot water. After centrifugation at 8000 rpm, the pellet was discarded and the water layer was partitioned by using equal volume of ethyl acetate twice. The collected ethyl acetate extracts were pooled together and were evaporated to dryness using rotary evaporator. The final residue was redissolved in 10 mL of distilled water and served as ethyl acetate (ESEA) extract.

### 2.3. Biochemical Screening of* E. singampattiana*

The basic biochemical screening tests were performed to identify various biochemical classes present in the endemic plant* E. singampattiana*. Modified procedure of Jeyaseelan and Jashothan [10] was used for the screening of biochemical and in brief the tannins were analysed by using 250 *µ*L of extract added with 500 *µ*L of distilled water and two drops of ferric chloride solution. The blue black coloration confirms the presence of tannins in the extract. Terpenoids test was made by using 500 *µ*L of extract added with 200 *µ*L of chloroform and 300 *µ*L sulfuric acid. Reddish brown color interface confirms the presence of terpenoids. For the saponins, 2 mL of extract was shaken vigorously to obtain a stable persistent froth and two drops of olive oil in the froth allowed for the formation of an emulsion, which indicated the presence of saponins. Flavonoids screening was made by adding 1 mL extracts with three drops of 1% ammonium solution and yellow color appearance indicated the presence of flavonoids. Cardiac glycosides were tested by adding 500 *µ*L of extract with 200 *µ*L of glacial acetic acid containing one drop of ferric chloride and the mixture was added with 100 *µ*L of concentrated sulfuric acid. Brown ring formation was observed. Phlobatannins were tested by adding extract (1 mL) boiled with 1% hydrochloric acid (1 mL) and observed for red precipitate. Alkaloid was tested by taking 1% hydrochloric acid (500 *µ*L) and 1.5 mL of extract in a test tube was treated with three drops of Meyer's reagent. A creamy white precipitate indicated the presence of alkaloids. Resins were tested by adding copper solution (1 mL) with extract (1 mL) and shaken vigorously. Green precipitate was observed for the presence of resin [10].

### 2.4. Total Polyphenol Content (TPC) and Total Flavonoid Content (TFC) Analysis

The total phenolic and flavonoid content were analysed by adapting the procedure of Maria John et al. [11] and Yoo et al. [12] using 96 well microplate reader (SpectraMax Plus384 Devises, CA, USA). For the extraction 0.1 g of leaf samples was extracted with 2 mL of different solvent (ESE, ESM, ESW, ESEA, and ESRW) by using sonication for 10 min followed by centrifugation at 8000 rpm for 10 min. The supernatant was vacuum-dried and dissolved in 1 mL methanol and served as extracts for the analysis of TPC, TFC, free radical scavenging activity, and secondary metabolite analysis using HPLC.

For the TPC analysis, 20 *μ*L of extract was added with 100 *μ*L of 0.2 N Folin-Ciocalteu's phenol reagent followed by addition of 80 *μ*L of saturated sodium carbonate. After 1hr incubation, the absorbance was measured at 750 nm and the phenolic content was measured against gallic acid as standard. In case of flavonoid analysis, 20 *μ*L of extract was mixed with 180 *μ*L of 90% diethylene glycol and 20 *μ*L of 1 N NaOH. The absorbance was measured at 515 nm, and naringin of 12.5 to 200 *μ*g/mL served as standard.

### 2.5. 2, 2′-Azinobis(3-ethylbenzothiazoline-6-sulfonic acid) Radical Scavenging Assay

ABTS analysis was performed by following the method of Lee et al. [13]. In brief 20 *μ*L of sample was mixed with 180 *μ*L of ABTS radical solution followed by 7 min of incubation under dark condition and the absorbance was measured at 750 nm.

### 2.6. Chelating Effects on Ferrous Ions

The metal chelating activity of the different extracts was measured by adding 20 *µ*L of extracts with 10 *µ*L of FeCl_2_ (1 mM) and 170 *µ*L of ferrozine (5 mM) [14]. The mixture was incubated for a period of 30 min and the absorbance was measured at 562 nm. Methanol served as control and all the above mentioned activities were calculated based on the difference between control and sample.

### 2.7. Phosphomolybdenum Activity

Modified procedure of Prieto et al. [15] was used for the detection of phosphomolybdenum activity of the different extracts. In brief 0.1 mL of the extract was mixed with 1 mL of reagent solution (0.6 M sulfuric acid, 28 mM sodium phosphate, and 4 mM ammonium molybdate) followed by incubation in a boiling water bath at 95°C for 90 min. The samples were measured at 695 nm and the antioxidant capacity was expressed as equivalents of *α*-tocopherol (mg/g of extract).

### 2.8. Primary Metabolite Extraction

Two different extraction procedures were checked for the primary metabolite analysis. Shade-dried samples of 100 mg were extracted with 1 mL of methanol : water : chloroform at the ratio of 2.5 : 1 : 1 (ES) and hot water (ESW) containing norvaline as an internal standard. The mixer was agitated with Retsch ball mill at 30/s followed by sonication (10 min). After centrifugation at 5,000 rpm at 20°C for 8 min, 600 *µ*L of supernatant was separated and was made up to 1 mL by adding 400 *µ*L of water. From this extract 400 *µ*L of the samples was separated and vacuum-dried followed by derivatization. 200 *µ*L of methoxyamine hydrochloride in pyridine (20 mg/mL) was added to the extract followed by incubation (90 min) at 30°C. To this mixer 100 *µ*L of N,O-bis(trimethylsilyl)trifluoroacetamide (BSTFA) containing 1% trimethylchlorosilane (TMCS) was added and was incubated at 37°C, for 30 min [11].

### 2.9. Primary Metabolites Analysis by Gas Chromatography-Mass Spectrometry (GC-MS)

The primary metabolite differences with different extraction procedures were studied by using Shimadzu GC-MS system (QP-2010 SE) with an autoinjector (AOC 20i). Rxi-5silMS capillary column (30 m length × 0.25 mm i.d × 0.25 *µ*M film thickness) was used for the analysis. The injector temperature was set at 250°C with the injection volume of 1 *µ*L. The oven temperature program starts from 80°C for 2 min followed by 300°C from 2 to 15 min with 10°C/min hold and was finally held for 3 min. The MS ionization was performed at −70 volts and the mass range was set at 50 to 600 m/z with 10 spectra per second acquisition rate.

### 2.10. Secondary Metabolite Analysis by High-Performance Liquid Chromatography (HPLC)

Agilent HPLC (1100, USA) system equipped with diode array detector (DAD) was used for the detection of metabolites. The binary gradient system of water (mobile A) and acetonitrile (mobile B) containing 0.1% formic acid served as mobile phase. The gradient flow starts with 10% B and continued for the 5 min followed by an increase up to 90% at 30 min. From 30–35 min 100% B was maintained followed by 10% B at 40 min. The flow rate was set at 1 mL per min and the detector was set at 254 nm for the detection of the secondary metabolites. Total of 40 individual standards were run prior to sample analysis and based on the retention time the metabolite identification was done with the samples.

### 2.11. Data Analysis

The data obtained from GC-MS were converted into netCDF (∗.cdf) formats by saving the file in AIA (Andi) file for the ∗.cdf conversion. Using metAlign, the CDF files underwent preprocessing, peak extraction, retention time correction, and alignment and the final data were exported to Microsoft Excel format (Microsoft, Redmond, WA, USA). This file was used for the multivariate statistical analysis using SIMCA-P software 12.0 (Umetrics, Umeå, Sweden). Based on the *P* value statistics and variable importance in projection (VIP) value the metabolites contributing significant variation between the two extraction procedures were identified. The partial least squares discriminant analysis (PLS-DA) from SIMCA-P software was not presented in this paper. The identified metabolites peak areas of GC-MS and HPLC were log_10_ followed by using Statistica 7 software; the levels were compared by box-whisker plot analysis.

### 2.12. Correlation Studies

The individual secondary metabolites area identified by HPLC was log_10_ compared with antioxidant activities. IBM SPSS Statistics 20 (SPSS Inc., Chicago, IL, USA) software was used for pairwise metabolite-antioxidant effects correlations analysed by Pearson's correlation coefficient test. The resulting Pearson's correlation coefficient values were plotted with heat map using MEV software version 4.8 (multiple array viewer, http://www.fm4.org/) and compared.

The whole experiment was repeated twice with proper replicates (*n* = 6) and the results were statistically analysed. Duncan's multiple-range test using SPSS software (SPSS Inc., Chicago, IL, USA) transformed and *P* value statistics from Statistica 7 were performed for the significant variation between the extracts.

### 2.13. Cells and Virus

Marc-145 cells were diluted to 2 × 105 cells/mL with 10% DMEM, seeded in 96-well plates, and incubated at 37°C in a 5% CO_2_ incubator. Porcine respiratory and reproductive syndrome virus (PRRSV) was propagated in Marc-145 cells. The tissue culture infectious dose 50 (TCID50) for the virus was determined by the Reed-Muench assay. Cell monolayers were treated with increasing concentrations of the appropriate extracts at the time of infection with 0.1 m.o.i. of PRRSV. The plates were then incubated at 37°C and cytopathic effect (CPE) was monitored at regular intervals. When CPE in the negative control reached 80%–90% compared with Marc-145 cells control, the cell viability was determined by the SRB Assay.

## 3. Results and Discussion 

### 3.1. Biochemical Screening of* E. singampattiana*

Basic biochemical screening of the endangered plant* E. singampattiana* was performed and presented in Table 1. Flavonoids were observed with all the extracts whereas alkaloids were not present with ESEA extracts. Tannins are confirmed with all the extraction except in ESEA partitioned samples. This plant was not confirmed for its saponin content whereas cardiac glycosides, resins, and phlobatannins were confirmed only with ESE and ESM extracts. Wang et al. [16] reported that the alkaloids were highly extractable in water extract and the extractability of ethyl acetate was poor due to its polarity. Due to the solvent polarity, alkaloids were not confirmed with ethyl acetate extract.

### 3.2. TFC and TPC Content of the Extracts

**Figure 1:**
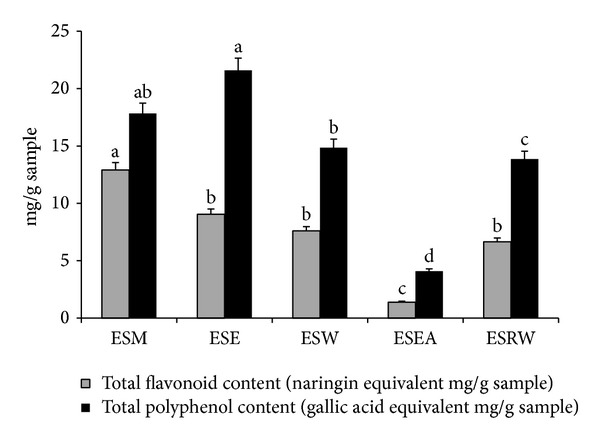
Total flavonoid and polyphenol content of an endangered plant* Eugenia singampattiana* leaf extracts ESE: ethanol; ESM: methanol; ESW: hot distilled water; ESEA: ethyl acetate; ESRW: water collected after ethyl acetate partitioning.

**Figure 2:**
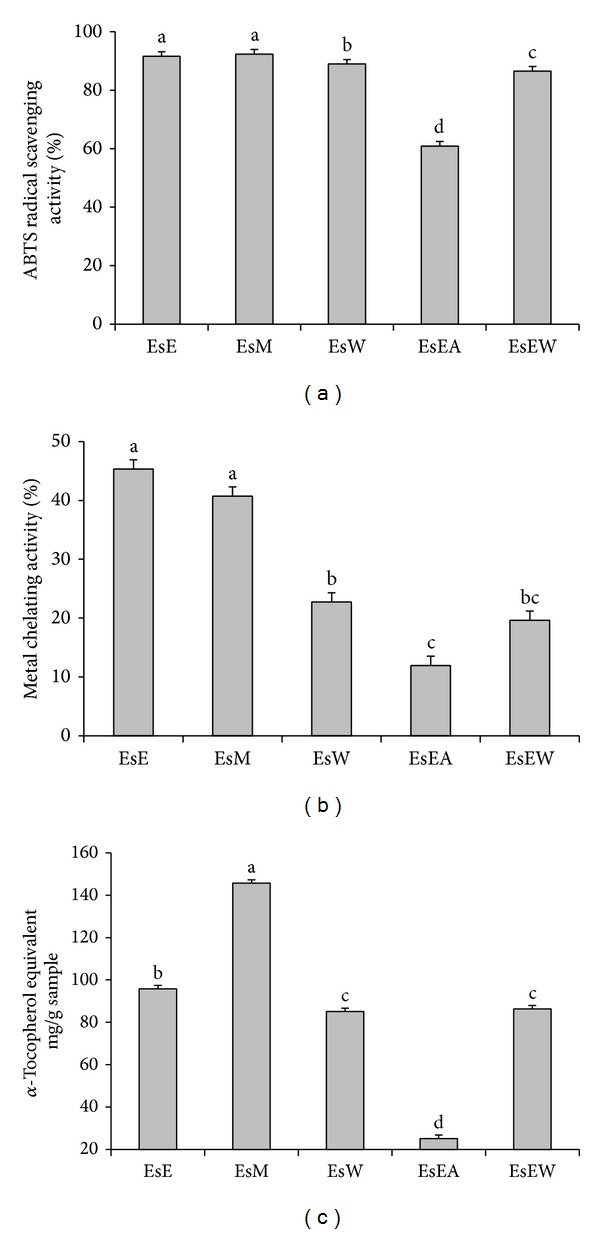
Free radical scavenging potentials of* Eugenia singampattiana* leaves extracted by various solvents. (a) ABTS; (b) metal chelating activity; (c) phosphomolybdenum. EsE: ethanol; EsM: methanol; EsW: hot distilled water; EsEA: ethyl acetate; EsRW: water collected after ethyl acetate partitioning.

Total flavonoid and polyphenol content of the different extracts were presented in Figure 2. Among the extracts, ESM extracts showed high flavonoid content (12.91 mg/g), whereas polyphenol content was high with ESE extract (21.58 mg/g) followed by methanol extract (17.84 mg/g). The ESEA extract showed poor content in their TFC (1.39 mg/g) and TPC (4.09 mg/g) content in the meantime; the flavonoid content of RW showed high total flavonoid and phenolic content than that of ESEA extract (Figure 1).

Ethanol, methanol, water, and acetone are commonly used solvent for the extraction of flavonoids from plant. According to Upadhyay et al. [17], the methanol was the best solvent for flavonoids extraction and the same result was observed with the present study. In both the analysis the metabolites were low with ESEA extracts revealed low level of compounds; hence, this partitioning method limits the extraction of phenolic, flavonoid compounds due to its saturation point, and polarity in the extraction of metabolites. Moreover the structure of the flavonoids is also one of the major reasons which affect the dissolvability by ethyl acetate and hence it requires repetitive extraction with the same solvent [18].

### 3.3. Antioxidant Potential of the Extract

The ABTS, metal chelation, and phosphomolybdenum activity of the different extracts were compared and presented in Figure 2. Comparing the antioxidant activity of various extracts, ABTS activity was high with ESE and ESM extracts followed by ESW extract. The metal chelating activity and phosphomolybdenum activity showed high variation between the extracts. Metal chelating activity was high with ESE, but the ESM extracts were on-par with ethanol extract statistically. Moreover the ESW extracts showed similar content and ESEA extract was found to be least among the scavenging activities. In case of phosphomolybdenum activity, ESM extract was found to be high followed by the ESE extract.

Antioxidant activity study by using different chemical substance has different mechanisms and for the study of antioxidant potential of the plants required different type of antioxidant test which shows different mechanisms is required to confirm the potentiality of the plants [19, 20]. Based on the present study it was clear that the extraction solvent contains different levels of phenolics and flavonoid content reflecting in their antioxidant potential. Moreover the antioxidant potential of the plants not only depends on the flavonoid content but also on the types of flavonoids and their bioavailability, for example, the flavan-3-ol such as EGCG and EGC has higher free radical scavenging activity than that of EC [21–23].

### 3.4. Primary Metabolite Variations from Different Extraction Procedure

**Figure 3:**
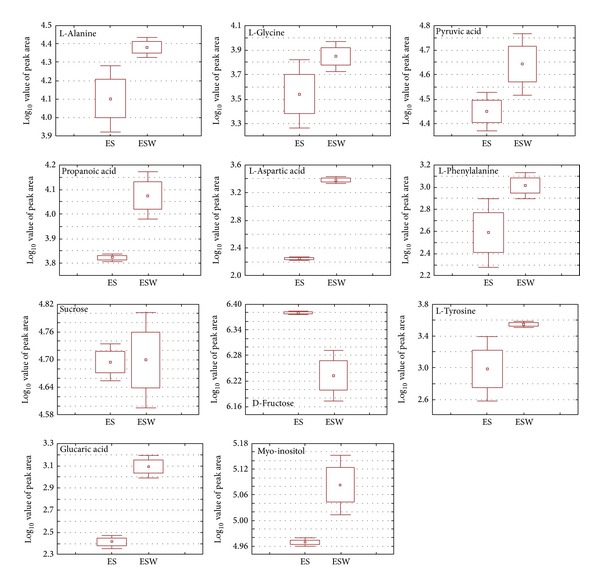
Box-whisker plot analysis of primary metabolites extracted by two different methods analysed by GC-MS. ES: extracted with methanol : chloroform : water; ESW: hot water extract.

The endemic medicinal plant* E. singampattiana* was not analysed for its primary metabolite such as organic acids and amino acids content; an attempt was made to find primary metabolite variation among different extraction procedure by using GC-MS. Based on the VIP values from the PLS-D Aanalysis using SIMCA P software the metabolites significantly causing variation among the extracts were identified and presented. Totally 11 primary metabolites significantly varied between the two extracts were identified based on MS fragmentation, library, and standards. The particulars of the metabolites along with its retention time and MS fragmentations were presented in Table 2. Most of the metabolites such as L-alanine, glycine, pyruvic acid, propanoic acid, L-aspartic acid, L-phenylalanine, sucrose, L-tyrosine, glucaric acid, and myo-inositol were high with ESW extract, whereas D-fructose was high with ES extract (Figure 3).

### 3.5. Secondary Metabolite Variations from Different Extraction Procedure

**Figure 4:**
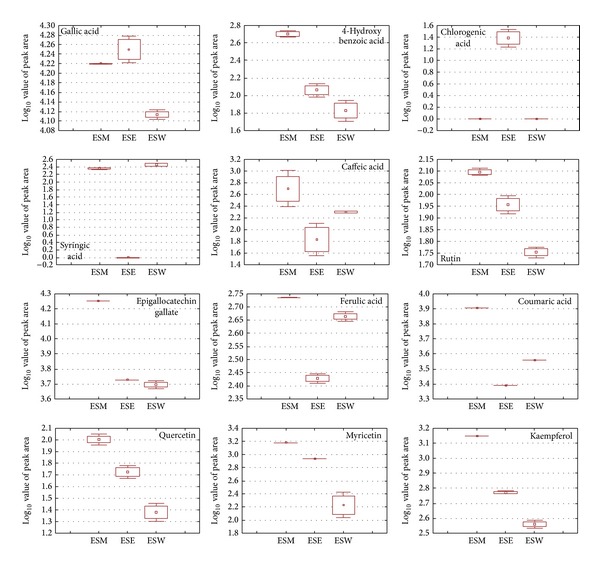
Box-whisker plot analysis of individual secondary metabolites causing variation between the extract analysed by HPLC. ESM: methanol extract; ESE: ethanol extract; ESW: hot water extract.

Based on the antioxidant potential it was clear that the antioxidant activity was high with ESM, ESE, and ESW and hence these three solvents were tested for their secondary metabolite variation using HPLC. A total of 12 secondary metabolites were identified based on standards and the particulars were presented in Table 3. Gallic acid, chlorogenic acid, and syringic acid content was high with ethanol extracts. Whereas all other metabolites like 4-hydroxybenzoic acid, caffeic acid, rutin, epigallocatechin gallate, ferulic acid, coumaric acid, quercetin, myricetin, and kaempferol content were high with methanol extraction (Figure 4). Among the different extraction solvents ESW showed lower quantity of metabolites. Ferulic acid content was high with water extract than that of ethanol extract and less than methanol extract as observed. This metabolic variation directly influences the antioxidant potential of different extraction methods.

Ethanol and methanol were mostly used for the metabolic profiling of medicinal plants. Based on plants, metabolic types, these solvent potential will change. Sultana et al. [24] reported that the phenolic and flavonoid content of various medicinal plants were higher with methanol than that of ethanol extract. Based on the polarity of the molecules the dissolving ability changed between metabolites in different solvent system and hence lowered percentage of water in ethanol and methanol extract resulted in high yield in terms of TPC and TFC content [24]. But in the present study the total flavonoid content was high with methanol extract because it was noticed from the HPLC analysis that in most of the content flavonoid molecules extracted were higher than ethanol extract, whereas the total phenolic content was high with ethanol extract because the phenolic acids such as gallic acid, chlorogenic acid, and syringic acid levels are more in the ethanol extract. Previous results of Pavendan and Sebastian [25] also demonstrate that the methanol extract of* E. singampattiana* showed high antimicrobial activity against various bacterial strains. Similarly the flavonoid content of methanol extract showed high antioxidant activity in the present study. According to Yilmaz and Toledo [26] the phenolic acids showed less antioxidant activity when compared to flavonoids. The fact that the flavonoid molecules such as epigallocatechin gallate, quercetin, rutin, myricetin, and kaempferol are highly correlated with antioxidant activity was observed from the present study evidently proving that the antioxidant potentiality was highly influenced by the flavonoid content of the plants.

Methanol : chloroform : water was used for the extraction of primary metabolites from soybean and resulted most of the organic acids, sugars, and amino acids were detected by GC-TOF-MS [11]. But in the present study hot water extract resulted in higher content of primary metabolite than that of solvent extraction. Studies of Kala et al. [9] revealed that the ethanolic extract analysed by GC-MS resulted in terpenoids and ketones were abundantly present in this plant.

### 3.6. Metabolic Correlation with Antioxidant Activity

**Figure 5:**
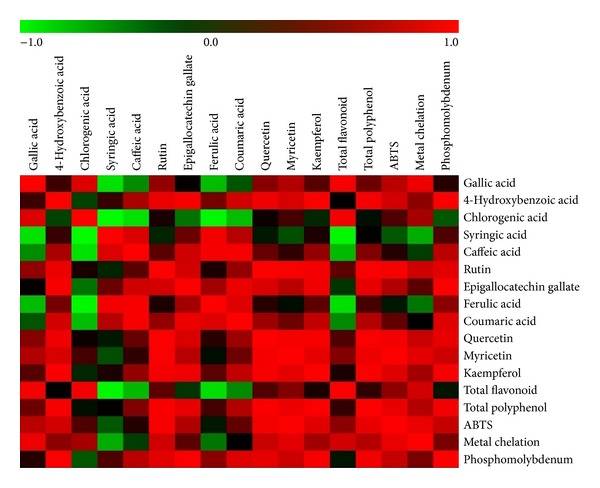
Correlation of secondary metabolites with antioxidant potential of* E. singampattiana* leaf extracts.

Pairwise correlation between the individual metabolites identified by HPLC and the antioxidant potential was compared and presented in Figure 5. The metabolic correlation with antioxidant activity showed that most of the compounds have positive correlation with ABTS, metal chelating, and phosphomolybdenum activity. Syringic acid, ferulic acid, and caffeic acid showed negative correlation with metal chelating activity. When comparing, the ABTS and phosphomolybdenum activity against metabolites syringic acid and chlorogenic acid were negatively correlated. In case of ABTS, rutin, quercetin, myricetin and TPC were having high positive correlation whereas gallic acid and myricetin in case of metal chelating activity and 4-hydroxybenzoic acid, epigallocatechin gallate, quercetin, kaempferol and TPC in case of phosphomolybdenum activity resulted in high positive correlations. The metabolites like 4-hydroxybenzoic acid, caffeic acid, rutin, epigallocatechin gallate, ferulic acid, coumaric acid, quercetin, myricetin, and kaempferol content were highly changed based on their extraction solvent and hence the antioxidant activity of the extracts has difference in terms of their scavenging property.

### 3.7. Antiviral Assay

**Figure 6:**
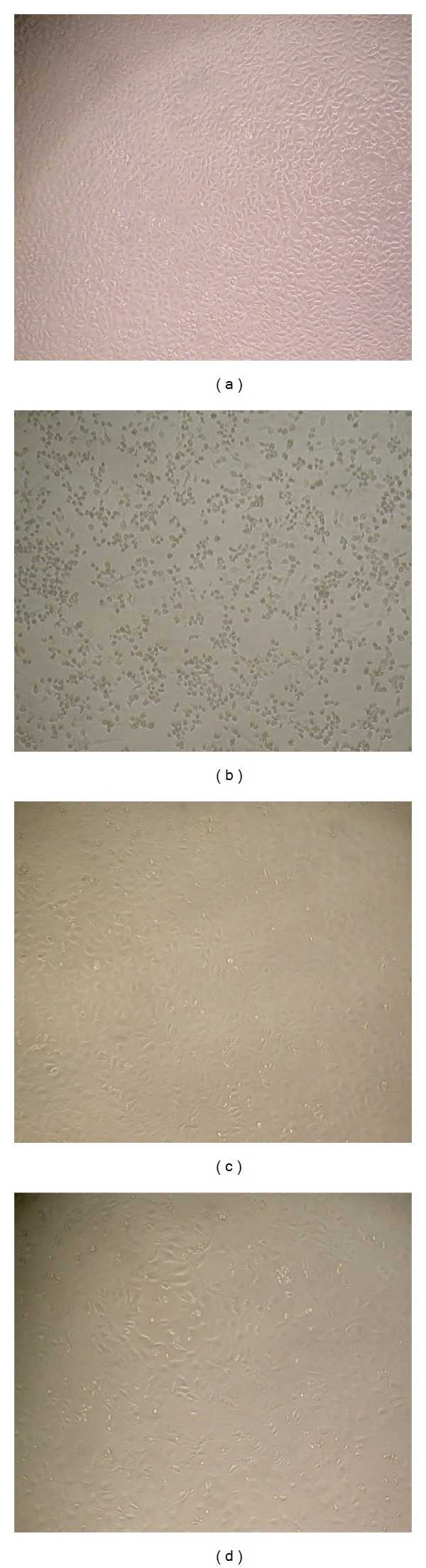
Anti-PRRSV activity of the two compounds on Marc-145 cells. (a) Cell control (only the Marc-145 cells); (b) Marc-145 cells infected with PRRSV (without plant extracts)support the virus replication and formation of CPE; (c) PRRSV treated with water extract showed antiviral activity (100 *µ*g of water extract treated cells inhibits replication of virus); (d) PRRSV treated with water extract showed antiviral activity (25 *µ*g of methanol extract treated cells inhibits replication of virus).

The anti-PRRSV activity of the compounds and cytotoxicity were evaluated with different concentrations (25 *µ*g to 1000 *µ*g) of compounds for both the extracts. The Marc-145 cells served as control (Figure 6(a)) and the Marc-145 cells infected with PRRSV are presented in Figure 6(b). To identify the antiviral activity of the extracts, the cells treated with water extract and methanol extracts were presented in Figures 6(c) and 6(d), respectively. The anti-PRRSV activities of both extracts have slight differences. Water extract shows an effective inhibitor of PRRSV at above 100 *µ*g and about 75% of inhibition observed at 50 *µ*g. However, methanol extract shows effective inhibition at above 10 *µ*g. The Marc-145 cells show morphology changes, when it is treated above 500 *µ*g. Therefore, the above results revealed that 75 to 100 *µ*g is an efficient activity of water extract and 25 *µ*g is an efficient activity of methanol extract. The results demonstrated significant and reproducible antiviral activity of both extracts against PRRSV.

PRRS virus causes porcine reproductive and respiratory syndrome (PRRS) found to be an acute infectious disease threatening swine production worldwide and causes even death by respiratory disorder in piglets and young pigs [27]. Spread of this virus is currently devastating the swine industry globally and causes significant economic losses [28]. Even though the vaccines are available to control the viral disease the efficacy and safety precautions forced researches to develop new vaccines or medicines and still the effective control of this virus was unknown [29]. The transgenic plant proteins showed high efficacy against the virus as reported by Hu et al. [30]. Previous studies illustrate that the water extracts were effective against viral pathogens [31, 32] but because of the high flavonoid content in ESM extract the antiviral activity was more when compared with ESW. The water extracts against pathogenic virus were studied before and showed good results. Plants possess various metabolites which naturally have antiviral activity and hence herbal products are effectively used for antiviral activity [33]. Identifying the plants which shows high viral activity can be a good source for the developments in the viral studies.* E. singampattiana* is one among them, which extremely is good antiviral activity against the PRRSV. Studying the plant further will be helpful for further development in the pharmaceutical industry for the wellbeing of human kind.

Plants are the rich source for new therapeutic compound development against H1N1 since this viral disease was spared worldwide.* E. singampattiana* showed good antimicrobial activity against PRRSV and particularly methanol extract showed that with low quantity (25 *µ*g) the activity was high. The methanol extract possesses higher extractability of 4-hydroxybenzoic acid, caffeic acid, rutin, epigallocatechin gallate, ferulic acid, coumaric acid, quercetin, myricetin, and kaempferol when compared to other extraction solvents. The antioxidant activity and metabolic correlation also suggest that the methanol extract was good for the profiling of* E. singampattiana*. This information will give some idea for the researchers to explore the antimicrobial compound from the plant resulting in the development of new pharmaceutical products.

## Figures and Tables

**Table 1:** Basic phytochemical screening of endangered plant *Eugenia singampattiana* extracts.

Phytochemicals	ESE^@^	ESM^#^	ESW^$^	ESEA^%^
Tannins	(+)	(+)	(+)	(−)
Terpenoids	(+)	(+)	(+)	(+)
Saponins	(−)	(−)	(−)	(−)
Flavonoids	(+)	(+)	(+)	(+)
Cardiac glycosides	(+)	(+)	(−)	(−)
Alkaloids	(+)	(+)	(+)	(−)
Resins	(+)	(+)	(−)	(−)
Phlobatannins	(+)	(+)	(−)	(−)

^@^Ethanol; ^#^methanol; ^$^hot distilled water; ^%^ethyl acetate; (+) presence; (−) absence of the phytochemicals.

**Table 2:** Particulars of the primary metabolites significantly contributing variations between the extracts analysed by GC-MS.

S. No	Rt^$^	MS fragmentation	Metabolite	*P* Value	Ref^@^
1	03.77	116, 73, 147, and 218	L-Alanine	6.29*E* − 02	Std^#^, lib^&^
2	04.17	73, 75, 79, 130, 93, and174	Glycine	1.55*E* − 01	Lib.
3	04.26	73, 147, 133, 59, 86, and 100	Pyruvic acid	8.58*E* − 02	Lib.
4	06.91	78, 147, 189, 292, 103, 133, and 205	Propanoic acid	1.03*E* − 02	Lib.
5	09.55	73, 232, 100, 147, and 306	L-Aspartic acid	4.00*E* − 05	Std., lib.
6	10.62	73, 218, 192, 100, 266	L-Phenylalanine	8.91*E* − 02	Std., lib.
7	20.67	361, 73, 217, 362, 271, 147, and 103	Sucrose	9.49*E* − 02	Std., lib.
8	13.67	73, 103, 307, 217, 147, 277, and 364	D-Fructose	1.24*E* − 02	Std., lib.
9	13.87	73, 218, 100, 147, and 267	L-Tyrosine	7.32*E* − 02	Std., lib.
10	14.35	73, 333, 147, 217, 277, and 103	Glucaric acid	6.00*E* − 04	Std., lib.
11	15.97	73, 217, 147, 305, 103, 265, and 367	Myo-inositol	2.97*E* − 02	Std., lib.

^$^Retention time; ^@^reference; ^#^standards; ^&^MS library.

**Table 3:** Particulars of the secondary metabolites significantly varied among the extracts analysed by HPLC.

S. number	Metabolites	Rt^$^	*P* Value	Ref^@^
1	Gallic acid	01.7	8.60*E* − 03	Std^#^
2	4-Hydroxybenzoic acid	09.5	1.70*E* − 03	Std.
3	Chlorogenic acid	09.6	9.07*E* − 02	Std.
4	Syringic acid	09.9	6.00*E* − 06	Std.
5	Caffeic acid	10.4	7.80*E* − 02	Std.
6	Rutin	11.4	2.40*E* − 03	Std.
7	Epigallocatechin gallate	11.6	6.00*E* − 05	Std.
8	Ferulic acid	12.7	5.00*E* − 04	Std.
9	Coumaric acid	12.8	4.00*E* − 07	Std.
10	Quercetin	15.4	4.40*E* − 03	Std.
11	Myricetin	26.2	1.07*E* − 03	Std.
12	Kaempferol	29.8	1.07*E* − 03	Std.

^$^Retention time; ^@^reference; ^#^standard.
